# What should we call mental ill health? Historical shifts in the popularity of generic terms

**DOI:** 10.1371/journal.pmen.0000032

**Published:** 2024-06-04

**Authors:** Nick Haslam, Naomi Baes

**Affiliations:** Melbourne School of Psychological Sciences University of Melbourne Parkville, Melbourne, Victoria, Australia; University of Luxembourg: Universite du Luxembourg, LUXEMBOURG

## Abstract

Substantial attention has been paid to the language of mental ill health, but the generic terms used to refer to it–“mental illness”, “psychiatric condition”, “mental health problem” and so forth–have largely escaped empirical scrutiny. We examined changes in the prevalence of alternative terms in two large English language text corpora from 1940 to 2019. Twenty-four terms were studied, compounds of four adjectival expressions (“mental”, “mental health”, “psychiatric”, “psychological”) and six nouns (“condition”, “disease”, “disorder”, “disturbance”, “illness”, “problem”). Terms incorporating “condition”, “disease” and “disturbance” became less popular over time, whereas those involving “psychiatric”, “mental health” and “illness” became more popular. Although there were some trends away from terms with medical connotations and towards more normalizing expressions, “mental illness” consolidated its position as the dominant term over the study period.

## Introduction

Terminology has long been a vexed issue in the domain of mental ill health. Blatantly stigmatizing colloquial expressions such as “crazy” and “lunatic” have been controversial for many years [1], and official diagnostic terms such as “schizophrenia” have also been denounced [2]. Some critics challenge the use of diagnostic terms in general, sometimes out of concern for the ill effects of labelling [3,4] and sometimes driven by a broader critique of medicalization [5]. Some writers who are comfortable with diagnostic terms criticize disease-first language (e.g., “schizophrenic person”) for reducing people to their illnesses, whereas others criticize person-first language (e.g., “person with schizophrenia”) because some people strongly identify with their diagnosis [6,7]. There is also lively disagreement about appropriate terminology for referring to users of mental health services, such as “patient”, “client”, or “consumer” [8].

Generic terms for mental ill health are one kind of terminology that has largely escaped systematic attention. These expressions serve as umbrella terms that refer to the class of specific conditions. Official psychiatric classifications such as the Diagnostic and Statistical Manual of Mental Disorders (DSM) and the International Classification of Disease (ICD) employ the term “mental disorder,” but laypeople and professionals alike use a wide array of other expressions. These are typically compound, involving an adjectival expression followed by a noun, the former stipulating how the latter applies to the specific domain of mental ill health. Among the most common adjective expressions are “mental”, “psychological”, and “psychiatric”, with some writers now preferring “mental health” despite the occasional clumsiness of three-word terms such as “mental health disorder”. Some of the most common nouns include “condition”, “disease”, “disorder”, “disturbance”, “illness”, and “problem”. All combinations of these adjectives and nouns can be encountered in everyday use.

These adjectival and noun expressions have distinctive connotations. As a generic term, “mental” implies that the common element of the phenomena of interest relates to the mind. Thesauri list “physical” and “somatic” as antonyms of “mental”, arguably suggesting a dualistic contrast between “mental” and “physical” conditions. “Psychological” has a similar connotation (“of, relating to, or occurring in the mind” in the Merriam-Webster dictionary) but with a clearer connection to a specific profession and field of study (“of or relating to psychology” in the same dictionary). “Psychiatric” relates the phenomena of interest to a particular medical specialty. Like “psychiatric”, and unlike “mental” and “psychological,” “mental health”, used adjectivally (e.g., “mental health problem"), invokes a health context but without referring to a specific profession or discipline.

The noun components of generic terms also carry differing meanings, varying in the extent to which they implicate a medical framing of mental ill health. “Disease” and “illness” are arguably the most clearly medical. According to the ‘Small World of Words’ word association norms [9], the terms are strongly associated with one another and with “sickness”. Conceptually, “disease” refers to an objective organic malfunction, an entity prototypically caused by an external pathological agent such as a bacterium. “Illness,” by contrast, refers to the subjective experience of a state of ill health. “Disorder” and “disturbance” are sometimes used as near synonyms of “disease” and “illness” but imply a functional impairment or aberration rather than a structural pathology. According to the Compact Oxford dictionary [10], “disorder” is “usually a weaker term than disease, and not implying structural change” (p.449). “Disturbance” is less tied to the health domain than “disorder”, primarily linked to “problem” and “disruption” in Small World of Words. “Condition” is more neutral than most of the other terms, capable of referring to positive and negative states of health. “Problem,” finally, implies a negative state–associated in the word association norms with words such as “solve” and “issue”–without any direct reference to health. In short, the components of generic terms vary widely in their linkage to health and medicine and in their implied normalcy.

Terminology of this sort is one aspect of what Berrios [11] calls “psychopathological language”, the systematic language that at any point in history is deemed appropriate for referring to the psychopathological domain. Many generic terms have been widely used in recent history. Berrios notes that “insanity” and “madness” were popular terms in the 19^th^ century. “Mental disease” became a popular expression early in the late 19^th^ and early 20^th^ century, featuring in the title of the *Journal of Nervous and Mental Disease*, the world’s oldest scientific monthly devoted to human behavior. “Mental disorder” became the preferred generic term in organized psychiatry when formal psychiatric classifications were developed, such as DSM’s first edition, published in 1952. The emerging preference for this term may have arisen because it did not presume a biomedical causation and side-stepped debates over the legitimacy of “mental illness” or “mental disease” [12,13]. More recently, terms whose connotations are even less medical have become popular, such as those including “mental health” and “problems.” The emergence of these expressions reflects a desire to destigmatize and normalize mental ill health, akin to the “euphemism treadmill” [14], whereby new terms replace those that have come to be seen as offensive or pejorative to ameliorate them. As a result of these terminological shifts, many generic terms are now in widespread circulation.

The implications of alternative generic terms are unclear and have attracted little research. Despite having different connotations, several alternative terms (“mental disorder”, “mental illness”, “mental health problem”) do not differ substantially in the range of phenomena to which they refer [15]. There is also little clear evidence of differential impacts on judgments of people with mental ill health. Szeto, Luong, and Dobson [16] found that undergraduate participants did not differ in their attitudes towards and desire for social distance from a person labelled as having a “mental disease”, a “mental disorder”, a “mental health problem”, or a “mental illness”. Similarly, Fox et al. [17] found no effects on a range of stigma measures when the terms “mental illness”, “mental health problem” or “psychological disorder” were used in a large sample of people with a history of mental illness. However, Lawson [18] found a greater desire for distance from a hypothetical person when they were labelled as having a “mental disorder” rather than a “mental illness” or “mental health condition.” As yet, no studies have examined other dimensions along which alternative terms might have differential implications, such as effects on professionals’ clinical judgments or on laypeople’s beliefs about causes and appropriate treatments. Although there may have been a trend away from directly medical terms towards more normalizing alternatives, there is as yet no evidence to suggest such a trend has had beneficial effects.

The previous empirical work on generic terminology reviewed above has compared four terms at most, and theoretical work has typically addressed the strengths and weakness of single terms (e.g., “mental illness” [19]). Studies have also restricted their focus to current usage of terms rather than how that usage has evolved over time. The present study therefore investigated historical trends in the popularity of a comprehensive set of generic terms. We examined the frequency of 24 terms relative to all terms and to one another over a 80-year period using two large English language text corpora. The study was primarily descriptive, aiming to characterize shifts in preferred terminology within society at large. However, we expected to find evidence of diminished popularity of more medical terms (e.g., those including “disease”) and rising preference for more normalizing terms (e.g., those including “mental health” and/or “problem”).

## Method

### Corpora

Two corpora were used to track the rise and fall of generic terms from 1940 to 2019. These corpora were chosen for their wide historical span, their very large magnitude, and their differing text sources. The first was derived specifically from books published across the Anglophone world, whereas the second includes text from diverse sources in the USA. If historical trends in terminology are robust, they should replicate across these two distinct corpora. The open-access repository contains all preprocessing scripts: https://osf.io/6egsz/

The Google Books corpus contained “books predominantly in the English language published in any country”, incorporating 361 billion words that appear over 40 times across the corpus from the 1500s onwards [20]. Frequency counts for specific terms were extracted using the `ngramr`package in R Studio [21], which facilitates direct access to the corpus, and annual total frequencies were downloaded from Google Books Ngram Viewer Exports. This study used the most recently compiled general English version of the corpus (eng_2019), which excludes low optical character recognition quality and serials. The Google Books corpus contains numerals, did not require preprocessing, and contained 1,423,515,352,830 tokens in the 1940–2019 period.

The second corpus is a combination of two closely related corpora: the Corpus of Historical American English (CoHA [22]) and the Corpus of Contemporary American English (CoCA [23]). CoHA contains ~400 million words from 1810–2009, drawn from 115,000 texts distributed across everyday publications (fiction, magazines, newspapers, and non-fiction books). CoCA contains 560 million words from 1990–2019 drawn from ~500,000 texts (extracted from spoken language, TV shows, academic journals, fiction, magazines, newspapers, and blogs). A similar merged CoCA/CoHA corpus has been used in previous research [24].

After merging, the combined corpus spanning 1810–2019 was processed following recommendations from Alatrash et al. [25] to clean it without compromising the qualitative and distributional properties of the data. This process included first excluding the special token “@”, which appears in 5% of the COHA corpus (introduced for legal reasons), malformed tokens that are possible artifacts of the digitization process or the data processing, and clean-up performed using the web interface (“&c?;”, “q!”, “|p130”, “NUL”), and removing escaped HTML characters (“(STAR)”, “<p>”, “<>”). Other symbols were excluded after manual inspection of the corpus (e.g., “//”, “PHOTO”, “(COLOR)”, “ILLUSTRATION”). Blogs were also excluded (“web” = 89,054 articles; “blog” = 98,788 articles) for not containing associated year data. Forty-one lines were removed for missing text data (3 fiction, 11 news, 25 magazines, 2 spoken text). The cleaned corpus was then lower-cased and punctuation (commas, periods, question marks) was removed. Numerals and function words were retained to mirror the Google Books corpus. The final combined corpus contained 931,569,490 tokens from 370,091 texts from academic articles (*n* = 25,418), fiction books (*n* = 30,497), magazines (*n* = 136,493), newspapers (*n* = 113,440), non-fiction books (*n* = 2,635), spoken language (*n* = 43,210) and TV shows (*n* = 18,398). The current study restricted the corpus period from 1940 to 2019 using 716,070,640 tokens from 330,970 articles. Although very large, the combined CoCA/CoHA corpus was therefore 0.05% the size of the Google Books corpus.

### Generic terms

We examined 24 generic terms (bigrams and trigrams) by combining four adjectival terms (“mental”, “mental health”, “psychiatric”, “psychological”) with six nouns (“condition”, “disease”, “disorder”, “disturbance”, “illness”, “problem”). The popularity of each term in each time period was examined as its prevalence as a share of all terms in that period.

The relative frequency of generic terms for mental ill health as a proportion of all terms was extracted annually for the Google Books corpus but by decade (i.e., 1940–1949, 1950–1959 etc.) for the combined CoHA/CoCA corpus in view of its smaller size and the relative sparsity of the generic terms. Three sets of historical trends in the popularity of the generic terms were examined in parallel for the two corpora. First, we examined the frequency of the generic terms collectively to assess whether these terms have changed in their overall popularity. Second, we examined the frequency of the alternative adjectival and then noun terms relative to one another, to evaluate which terms have risen and fallen in relative popularity. In these analyses, the frequency of an adjectival expression is summed across all nouns it combines with (e.g., “mental” = “mental condition” + “mental disease” + “mental disorder” + “mental disturbance” + “mental illness” + “mental problem”) and vice versa. Finally, we examined trends in the relative popularity of the 24 compound generic terms to determine which have risen and fallen in dominance.

## Results

**Fig 1:**
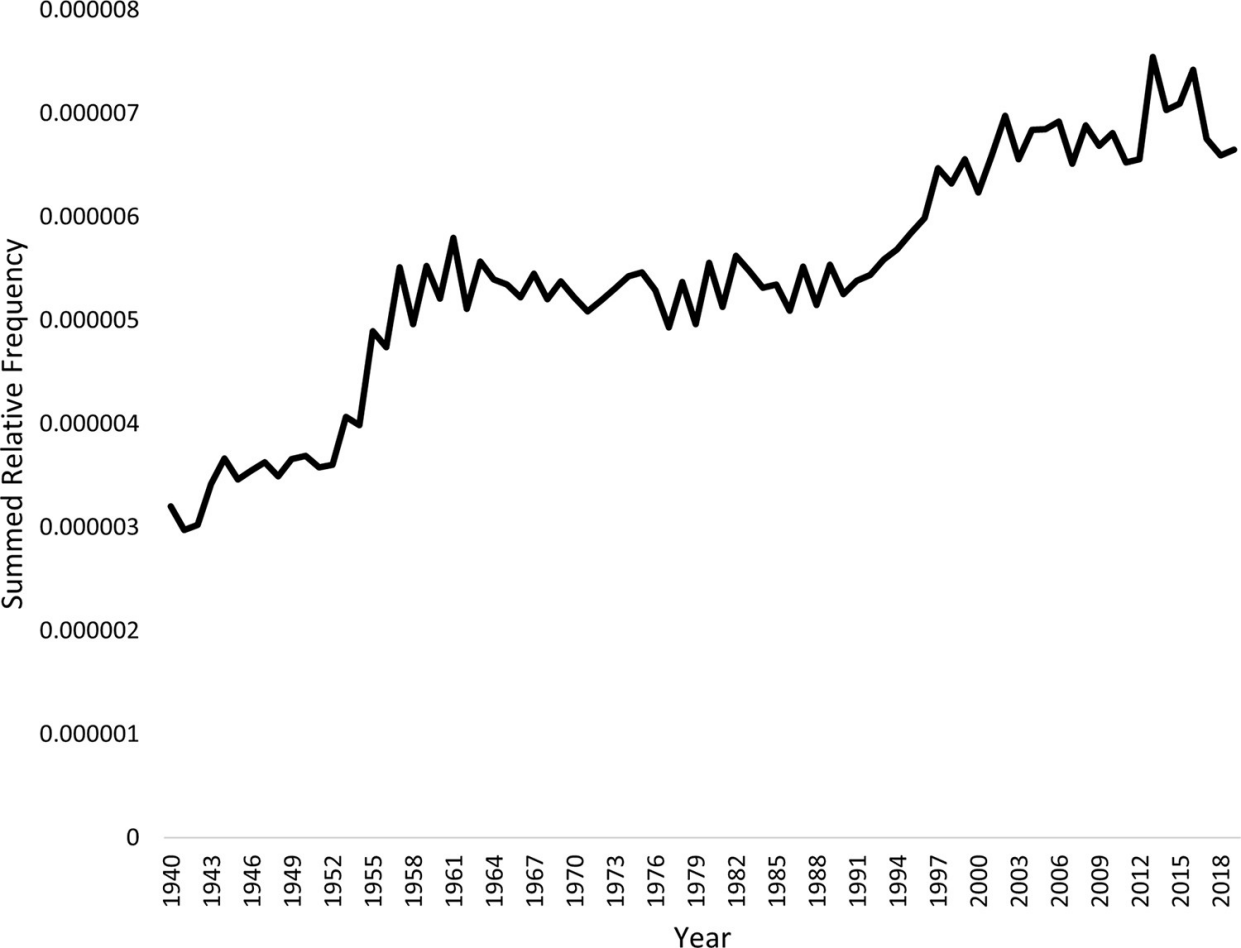
Summed relative frequency of the generic terms in the Google Books corpus.

**Fig 2:**
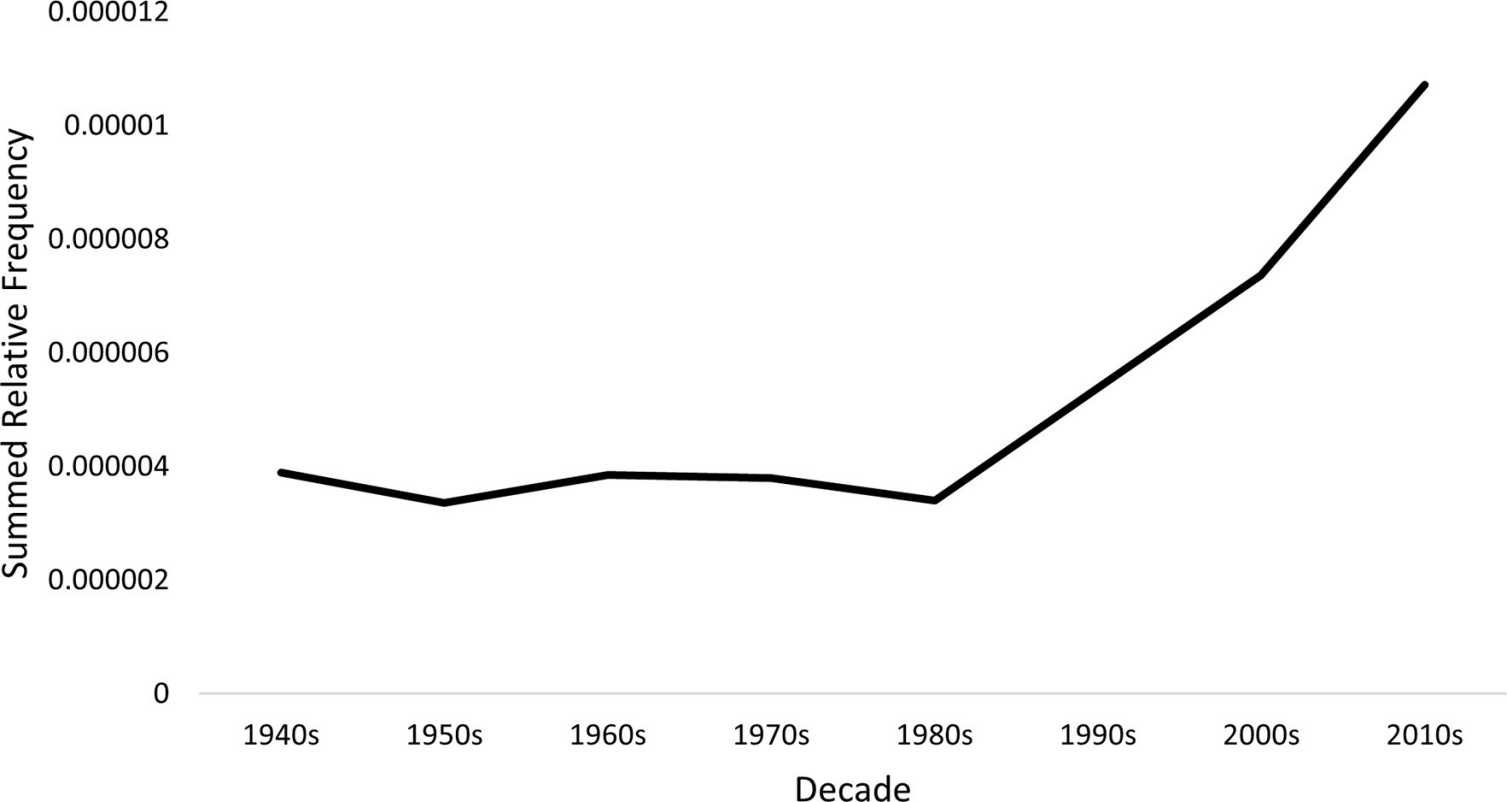
Summed relative frequency of all generic terms in the CoHA/CoCA corpus.

Figs 1 and 2 presents the combined relative frequency of the 24 generic terms in the Google Books and CoCA/CoHA corpora. Both corpora show strong upward trends, with the generic terms more than twice as prevalent in the most recent time period as at the beginning of the study period. This rise is consistent with the growing cultural salience of mental health and illness, and the rise of psychiatry, clinical psychology, and other mental health professions through the 20^th^ century and since.

**Fig 3:**
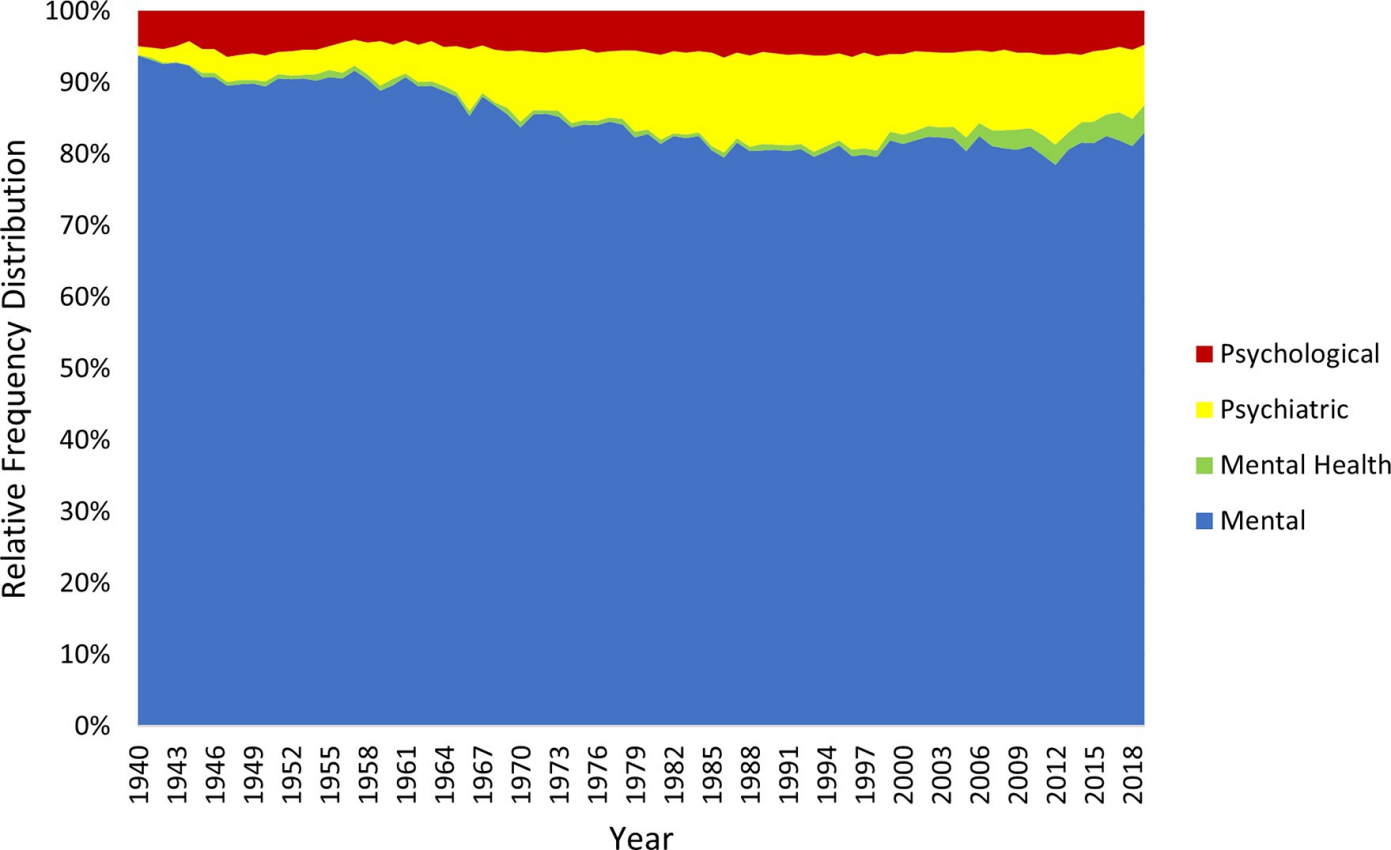
Relative popularity of adjectival terms in the Google Books corpus.

**Fig 4:**
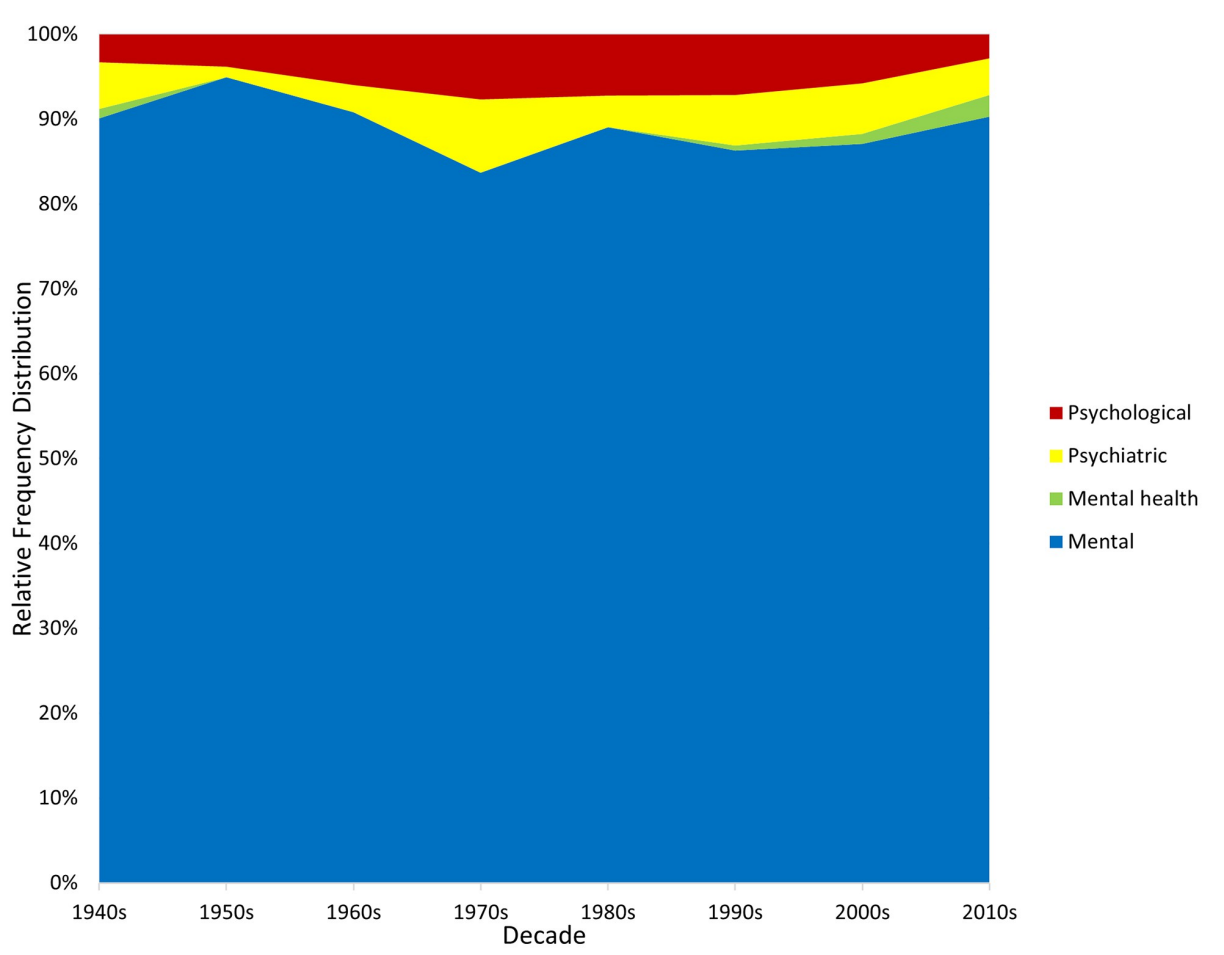
Relative popularity of adjectival terms in the CoCA/CoHA corpus.

Figs 3 and 4 displays the relative popularity of the adjectival components of the generic terms, each expressed as a percentage of all such expressions in each time period. The two corpora yield very consistent patterns. “Mental” is clearly the most prominent expression throughout the 80-year study period, reducing its share of all expressions only slightly. “Psychiatric” and “psychological” both emerge as increasingly popular adjectival expressions in the 1960s but then remain relatively stable. “Mental health” emerges in the 2000s but is always less popular than its alternatives.

**Fig 5:**
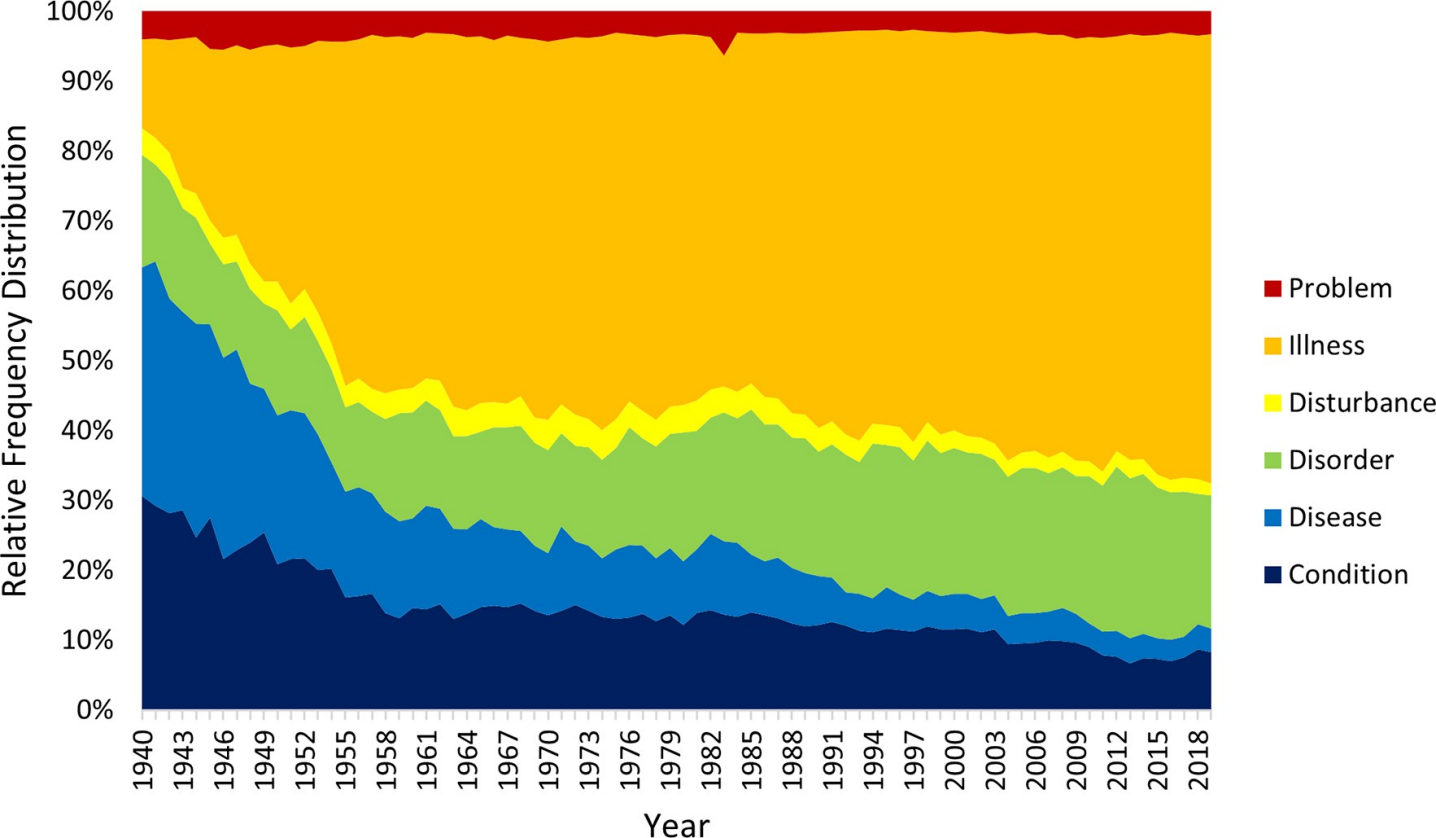
Relative popularity of noun terms in the Google Books corpus.

**Fig 6:**
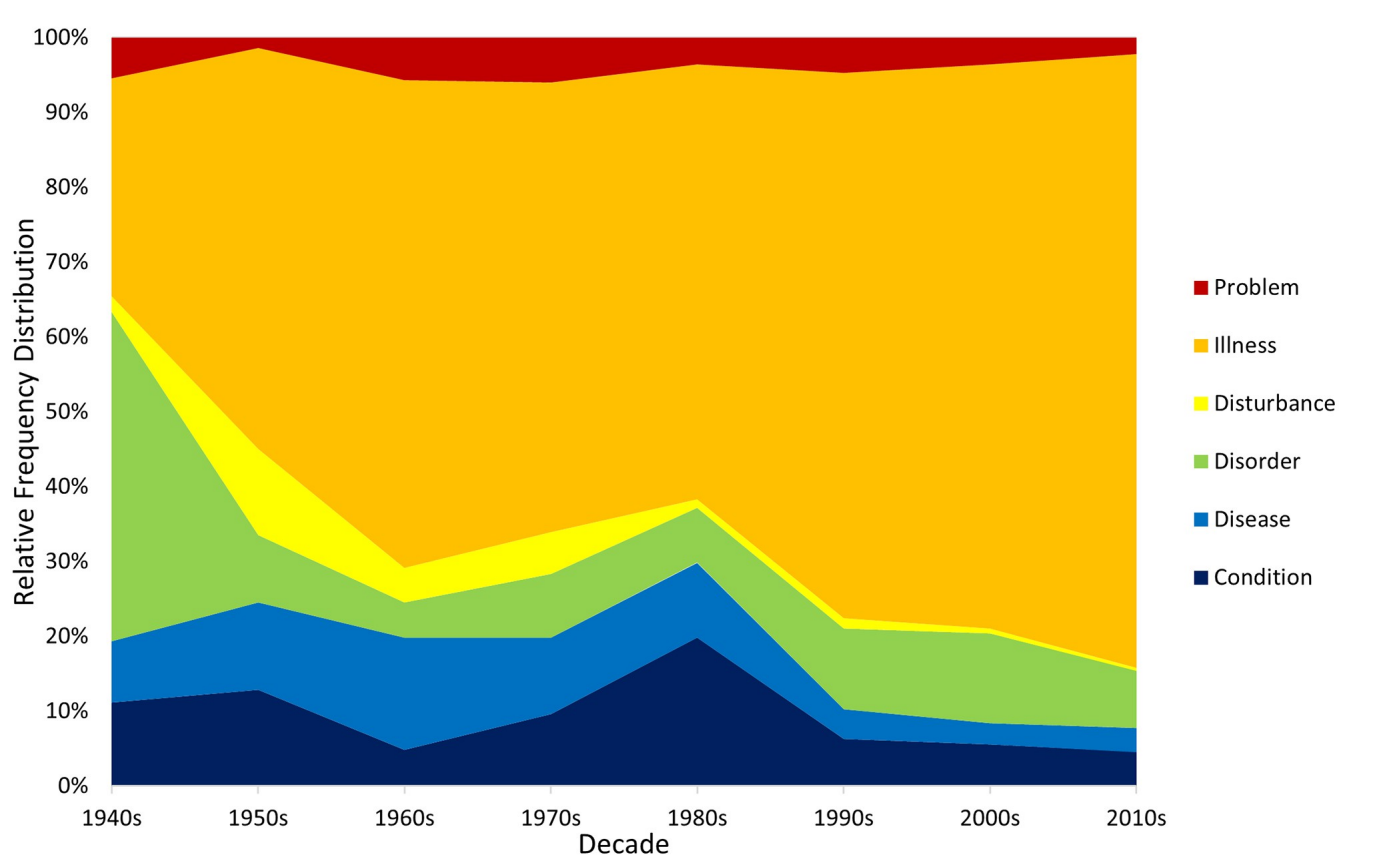
Relative popularity of noun terms in the CoHA/CoCA corpus.

Figs 5 and 6 show the corresponding trends for the noun expressions, which are again broadly consistent across corpora. Terms incorporating “disease” fell in popularity over time (especially in Google Books), those incorporating “problem” and “disturbance” were relatively unpopular but stable, and “illness” rapidly becomes the dominant noun term in the 1950s and steadily increased its popularity since then. The trajectories of the moderately popular “disorder” and “condition” terms are less clear, the former rising gradually in Google Books but falling across the first two decades in CoHA/CoCA, and the latter showing a general decline in recent decades.

**Fig 7:**
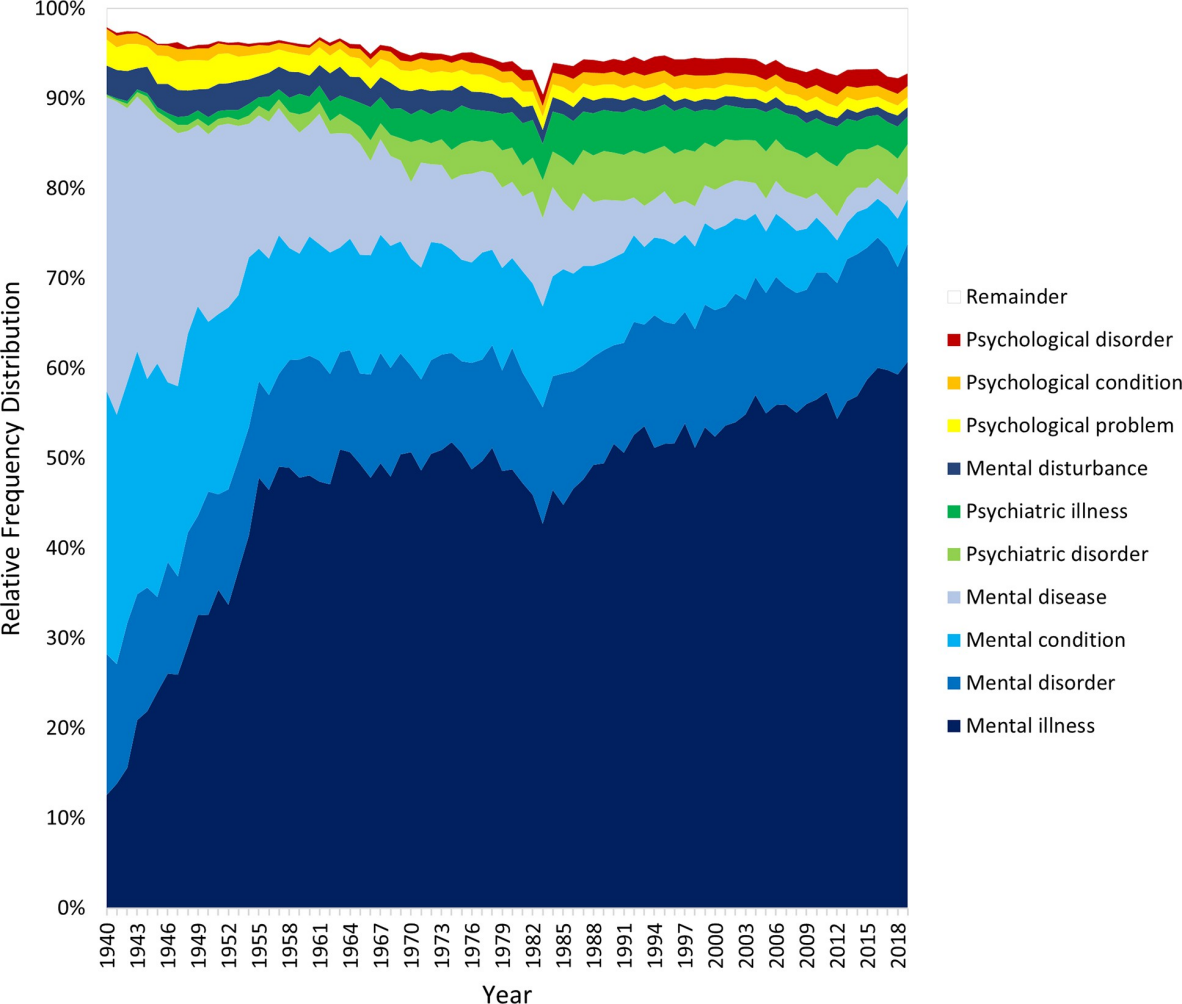
Trends in the top 10 combined terms in the Google Books corpus.

**Fig 8:**
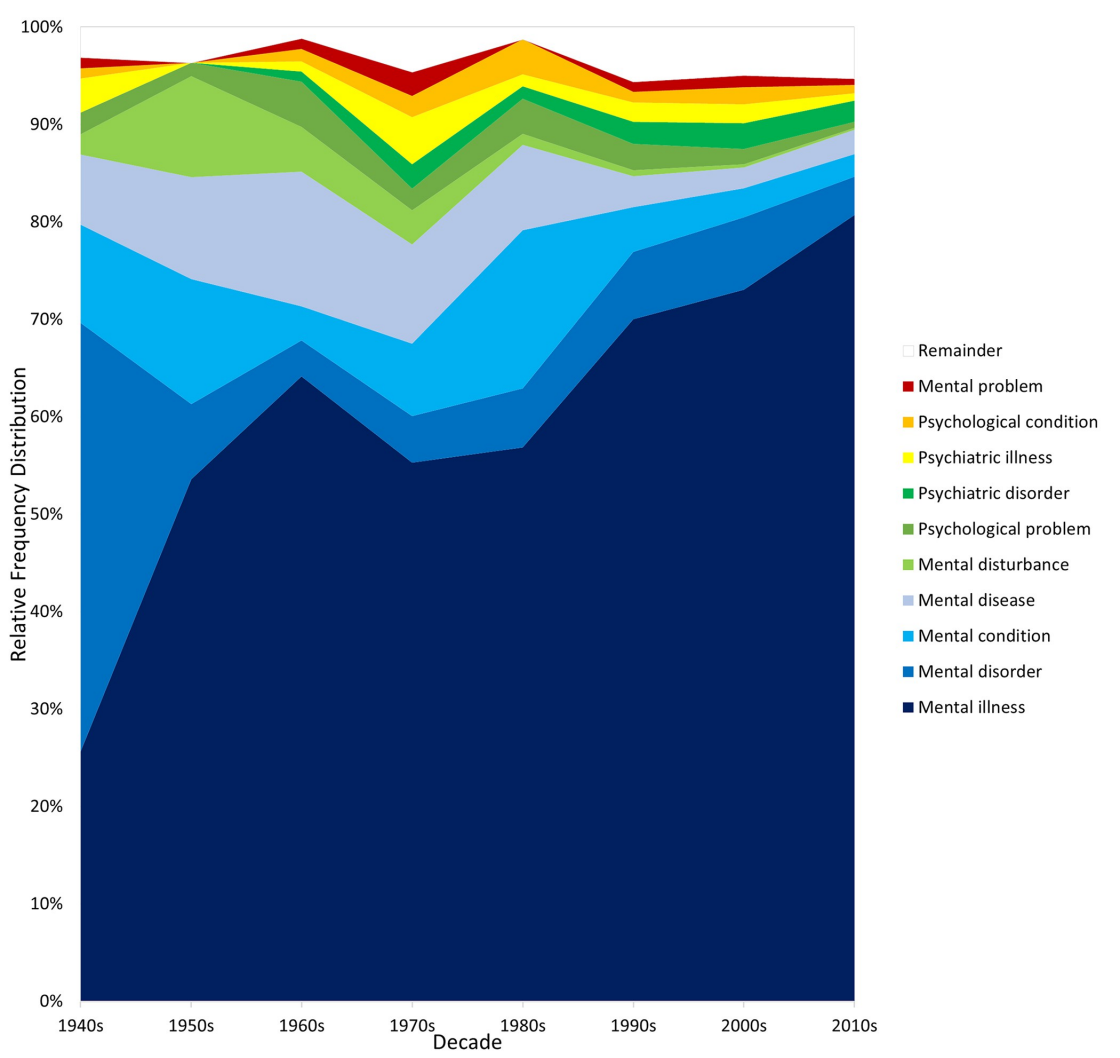
Trends in the top 10 combined terms in the CoHA/CoCA corpus.

Figs 7 and 8, finally, present the 10 most popular complete terms in each corpus, calculated based on their average relative frequency over the eight decades. The terms are ordered from bottom to top in average relative frequency, with the summed relative frequency of the 14 least popular terms represented by the white “Remainder” band. The two corpora yield highly convergent rankings, sharing nine of the top 10 terms and with their top four (“mental illness”, “mental disorder”, “mental condition”, “mental disease”) in identical order. “Mental illness” steadily rises to be the dominant generic term, “mental disease” steadily falls, “mental disorder” becomes a stable distant second, and two terms with “psychiatric” gain some ground in recent decades. However, most of the terms are of very low prevalence and demonstrate few meaningful historical shifts in popularity. Normalizing or de-medicalizing terms incorporating “problem” appear low in the top 10 once (Google Books) or twice (CoHA/CoCA), but those incorporating “mental health” do not.

## Discussion

In two large English language text corpora, we found consistent patterns in the popularity of a comprehensive set of generic terms for mental ill health. These patterns can be divided into those that are relatively stable over time and those that represent historical shifts. With regard to stable patterns, “mental” was overwhelmingly the most prevalent adjectival expression within generic terms throughout the 1940–2019 study period, with “psychiatric” and “psychological” far behind and “mental health” a very infrequent alternative. “Illness” was almost equally dominant as a noun expression within generic terms, with “disorder”, “disease”, and “condition” in a second tier and “disturbance” and “problem” rare. The relative unpopularity of “mental disorder” is surprising given the term’s ratification by influential psychiatric classifications. With regard to generic terms rather than their components, “mental illness” is easily the most prevalent in the study period, representing half or more of all uses of the 24 generic terms since the 1960s. Along with “mental disorder”, “mental condition” and “mental disease” it consistently accounts for more than 80% of all uses of generic terms throughout the study period in both corpora.

Patterns of change are also evident. Terms beginning with “psychiatric” and “psychological” made modest gains in popularity over time, as did those ending with “disorder”, whereas “disease” and “condition” tended to decline. Terms commencing with “mental health” grew steeply but from a very low base, and therefore did not feature among the most common generic terms. “Illness” consolidated its high popularity over time and “mental illness” rapidly rose to prominence, increasing its share of usage at least three-fold from 1940 to 2019. “Mental illness” rose most steeply from the 1940s to the 1960s at the apparent expense of “mental disease”, which fell steeply out of fashion during this period.

These patterns present a complex picture of the role of medical framing within generic terminology. Critics of medicalization have opposed terms they see as embodying a medical or disease model and proposed new terms to replace them. Their critique is often motivated by philosophical objections as well as the belief that the replacement terms will reduce stigma. Our findings offer some encouragement to these critics. Terms referring to “disease” have fallen from favor and those that include “psychiatric”, identifying mental ill health with a medical specialty, have not established a strong foothold. Normalizing terms such as “mental health problem”, which has no medical connotation and implies that mental ill health is an everyday dilemma to be solved, have become more prevalent in recent years.

Several findings point in the opposite direction, however. “Illness” may be less wedded to organic pathology than “disease”, but it is medical nonetheless and remains the dominant noun when referring to mental ill health. Its dominance, especially in the expression “mental illness”, has only increased in recent years, despite decades of criticism surrounding its legitimacy [13]. Less medically saturated terms that incorporate “mental health” or “problem” remain unpopular, at least as indexed by appearance in diverse forms of text, calling into question claims that “mental health” is increasingly used as a euphemism for “mental illness” [26]. Efforts to overhaul generic terminology have thus far not been effective in bringing about substantial change, and the rising prevalence and historical durability of “mental illness” suggests that altering public preferences for generic terms may be difficult. It could be argued that “mental illness” foregrounds subjective experience in its adjectival and noun components and should therefore be embraced rather than dismissed as medicalizing [19].

There has been very little systematic research on generic terms for mental ill health, so many possible avenues for future work are open. The corpora examined in the present study primarily represent written language generated by people outside the mental health professions, and it would be informative to assess preferences within and between these professions (e.g., clinical psychology, psychiatry, mental health nursing, social work). It would be equally informative to evaluate how professionals, laypeople and service users construe the differences in connotation between generic terms as well as their preferences among them, just as studies have examined preferences for alternative ways of referring to service users [8]. One informal exploration found that “some people prefer the phrase ‘mental illness’ as it emphasizes the seriousness of the conditions experienced by people; others prefer ‘mental health problem’ because they see it as less stigmatizing; others prefer mental ‘disorder’ as potentially encompassing both ‘problems’ and ‘illnesses’ while also acknowledging the non-medical dimension” [27] (p.46)]. A more systematic empirical investigation of understandings and preferences for generic terms is overdue.

Equally important is to establish whether generic terms have differential effects on perceptions of and by people experiencing mental ill health. Although vignette studies find few effects on stigmatizing attitudes [16,18], they are limited in quantity, in realism, and in the range of terms examined. No studies have explored whether generic terms have implications for how people with mental ill health perceive themselves (aside from Fox et al.’s [17] examination of self-stigma) or for how clinicians view them, including possible effects on the perceived durability, causation, or appropriate treatment implied by different terms. Is a condition described as a “psychiatric disease” likely to be perceived as more serious, organic, and suitable for pharmacological treatment than one described as a “mental health problem”? Examining the possible implications of different terms for how the general public and affected persons perceive and evaluate mental ill health should be a research priority.

Our study has several limitations. First, it only examines terms in English and its findings are unlikely to generalize to other languages. It would be worthwhile exploring shifts in preferred terminology in other linguistic and cultural contexts. Second, the study’s datasets ended in 2019 and there may have been significant changes since that time, during a period of intense attention to mental ill health. Future studies should examine ongoing terminological shifts. Third, the two corpora are drawn entirely (Google Books) or primarily (CoHA/CoCA) from written texts drawn from specific regions and therefore cannot be presumed to correspond to spoken language use or equally to all relevant geographical communities. More colloquial spoken language might employ different terms from written texts, or the same terms with significantly differing frequencies. The corpora are entirely (CoCA/CoHA) or predominantly (Google Books) based on U.S.A. sources, for example, and the extent to which our findings generalize across the Anglosphere is uncertain. Regrettably, addressing these possibilities may be challenging because corpora of comparable size and historical depth that collect spoken language or text from other regions may not exist. Fourth and more generally, while corpus studies enable powerful, large-scale quantitative analyses of language use, they do not allow for more nuanced analyses of connotational meaning or detailed studies of how words are understood or used differently in specific communities or contexts and possibly even replaced. Qualitative studies that illuminate these complexities would be valuable to complement our findings.

Debates over diagnostic labels, person- versus identity-first language, and appropriate ways of referring to people using mental health services reflect a conviction that language use in the field of mental health is profoundly important. The present study points to intriguing shifts in the use of generic terms for mental ill health, but it remains to be seen whether the implications of these terms are equally consequential.
